# Resolution of Pyoderma Gangrenosum During Adjuvant Breast Cancer Therapy

**DOI:** 10.3390/jcm14041320

**Published:** 2025-02-17

**Authors:** Abigail P. Lauder, Anita Nwiloh, Matthew Eximond, Robert E. LeBlanc, Alicia T. Dagrosa, Richard Barth, Mary Chamberlin, Shauna McVorran

**Affiliations:** 1 Department of Radiation Oncology and Applied Sciences, Dartmouth Hitchcock Medical Center, Lebanon, NH 03756, USA; 2 Meharry Medical College School of Medicine, Meharry Medical College, Nashville, TN 37208, USA; acnwiloh21@email.mmc.edu; 3 DeBusk College of Osteopathic Medicine, Lincoln Memorial University, Knoxville, TN 37752, USA; matthew.eximond@lmunet.edu; 4 Department of Pathology and Laboratory Medicine, Dartmouth Hitchcock Medical Center, Lebanon, NH 03756, USA; robert.e.leblanc@hitchcock.org; 5 Department of Dermatology, Dartmouth Hitchcock Medical Center, Lebanon, NH 03756, USA; alicia.t.dagrosa@hitchcock.org; 6 Department of Surgery, Dartmouth Hitchcock Medical Center, Lebanon, NH 03756, USA; richard.j.barth.jr@hitchcock.org; 7 Department of Medicine, Dartmouth Hitchcock Medical Center, Lebanon, NH 03756, USA; mary.d.chamberlin@hitchcock.org

**Keywords:** inflammatory skin disorder, neutrophilic dermatosis, wound healing, radiation therapy

## Abstract

**Background/Objectives:** Pyoderma gangrenosum (PG) is a rare neutrophilic dermatosis characterized by rapidly developing, painful ulcerative lesions. It exhibits pathergy, a phenomenon in which minor trauma or injury to the skin triggers an exaggerated inflammatory response. This leads to the development of new skin lesions or the worsening of existing ones. Treatment typically involves a combination of corticosteroids and immunosuppressive agents. However, even with effective therapy, the overall management of pyoderma gangrenosum remains challenging, and wound healing can be prolonged. The development of pyoderma gangrenosum after breast cancer surgery is rare, and its presence complicates the treatment of patients requiring additional oncologic therapy. In particular, the effect of radiation on these lesions is not well documented. Given the known skin toxicity of radiotherapy and its negative impact on wound healing, the use of adjuvant breast radiation raises significant concerns in this context. **Methods:** We present the case of a 66-year-old female with Stage IIB invasive ductal carcinoma of the left breast who developed postoperative pyoderma gangrenosum after breast-conserving surgery. The patient was treated with systemic corticosteroids and cyclosporine, and then subsequently underwent standard-of-care adjuvant chemotherapy and radiation. **Results:** During therapy, she demonstrated rapid resolution of her pyoderma gangrenosum without experiencing excess skin toxicity. **Conclusions:** While the literature on the direct application of radiation in pyoderma gangrenosum is limited, our case provides evidence supporting the safety of radiation therapy in oncologic cases complicated by this disease. In addition to receiving the benefit of adjuvant therapy for her breast cancer, our patient demonstrated an improvement in her postoperative PG with no adverse skin effects.

## 1. Introduction

Pyoderma gangrenosum (PG) is a rare inflammatory skin condition characterized by painful, rapidly progressing ulcerative lesions. Despite its name and clinical presentation, it is neither the result of an infection nor gangrene; PG lesions are sterile. While the underlying mechanisms of the disease are not fully understood, pyoderma gangrenosum is thought to be caused by dysregulation of both the adaptive and the innate immune system. It is characterized by a dense neutrophilic infiltration of the dermis, overlying epidermal ulceration, and necrosis, falling into a category of disorders known as neutrophilic dermatoses [1,2].

Pathologic findings of the disease are non-specific and clinical morphologies can vary. These morphologies include vesiculobullous, pustular, superficial granulomatous, and vegetative variants. Additionally, the histopathology of PG varies with the age of lesions. Biopsies of early lesions reveal dermal abscess in association with follicular and perifollicular inflammation. With progression, ulcer and superficial dermal necrosis become present. The inflammatory infiltrate may undermine the edge of the ulcer and extend to the subcutis. Perivascular lymphoplasmacytic inflammation and perivascular fibrin deposition may be present in the skin adjacent to the ulcer. Since no histopathologic finding is pathognomonic of PG, the diagnosis requires correlation with clinical history and morphology. Clinical context, including a history of associated conditions, pathology findings that belie an alternative etiology, and dermatologic examination are all required to make this diagnosis of exclusion.

Pyoderma gangrenosum is known to be associated with systemic disorders, particularly inflammatory bowel diseases such as Crohn’s disease and ulcerative colitis. It is also associated with hematologic malignancies and certain solid tumors, including those of the breast [3,4,5,6]. With or without the presence of these associations, PG exhibits pathergy, a phenomenon in which trauma to the skin can evoke or exacerbate lesions. Therefore, PG can be precipitated by surgery. Interestingly, pyoderma gangrenosum occurring after breast surgery specifically is notably rare. A systematic review identified only 56 reported cases of its development following breast procedures. Most of these cases were after breast reduction or reconstruction surgeries, and only six were reported after lumpectomy or mastectomy [7].

In addition to treatment of the underlying medical condition, treatment of pyoderma gangrenosum involves a combination of both corticosteroids and immunosuppression. Topical corticosteroids, systemic corticosteroids, and cyclosporine are common initial therapeutic approaches, and the response to treatment is assessed clinically. However, management can be challenging and the healing time of these ulcers can be very prolonged. In some instances, weeks to months are required to achieve complete resolution. For refractory cases, targeted therapy in the form of biologics has shown promise. The most common biologics employed as second-line therapy in the treatment of pyoderma gangrenosum include anti-tumor necrosis factor drugs (e.g., infliximab, adalimumab, and etanercept) [8].

The effects of chemotherapy and radiation therapy on active lesions are not well documented. This is especially concerning when considering administration of radiation therapy. Radiation not only has the potential to cause dermatitis, but is also known to disrupt skin repair mechanisms, leading to delayed or impaired wound healing [9]. This context underscores the importance of understanding how these factors can affect patient outcomes. Here, we present a unique case of pyoderma gangrenosum that developed after breast cancer surgery and showed significant and rapid healing during adjuvant therapy. Through this case report, we aim to contribute to the existing literature on this topic and provide insights into the potential interactions of PG with breast cancer therapy, specifically radiation therapy.

## 2. Detailed Case Description

Our patient is a 66-year-old female, non-smoker, with a past medical history significant for uncomplicated bilateral metachronous ductal carcinoma in situ (DCIS). This was treated with lumpectomy and adjuvant radiation on the right 16 years prior to this presentation and lumpectomy alone on the left 14 years prior to this presentation. The patient also has a history of familial polyposis syndrome (FAP) managed with a right hemicolectomy, which was complicated postoperatively by an unspecified necrotizing infection. The patient’s family history includes colon cancer and breast cancer in her mother, as well as breast cancer in three sisters and a paternal aunt. She has no known family history of pyoderma gangrenosum.

Annual surveillance mammograms were benign until many years after her prior DCIS treatments, when suspicious calcifications were noted in the patient’s posterior left breast. Diagnostic mammogram and ultrasound showed a 2.5 cm area of microcalcifications in the upper-outer quadrant of the left breast at 1 o’clock, 5 cm from the nipple. This imaging was read as BI-RADS 5 [10]. Biopsy was then performed, with pathology revealing a microinvasive carcinoma in a background of high-grade DCIS with comedonecrosis. Subsequent bilateral breast MRI demonstrated a 3.2 × 2.8 cm region of heterogenous and clumped non-mass enhancement at 9 o’clock, 3.3 cm from the nipple. There was no evidence of lymphadenopathy or metastatic disease.

She therefore underwent a left lumpectomy with sentinel lymph node biopsy. Final surgical pathology revealed multifocal, high-grade, invasive ductal carcinoma. The disease was 2.1 cm in its greatest dimension and was associated with high-grade DCIS with an extensive intraductal component, measuring 4 cm. The surgical margins were all negative and the excised lymph node was found to be negative for metastatic disease. Immunohistochemical and FISH staining of the surgical specimen showed ER positivity 1–10%, PR negativity, and HER2 negativity. The patient’s oncotype recurrence score returned at 58. Her pathologic prognostic staging per the AJCC 8th edition was pT2(m)N0(sn)M0, Stage IIB [11]. She has a strong family history of breast cancer, and genetic testing revealed a BARD1 mutation.

**Figure 1:**
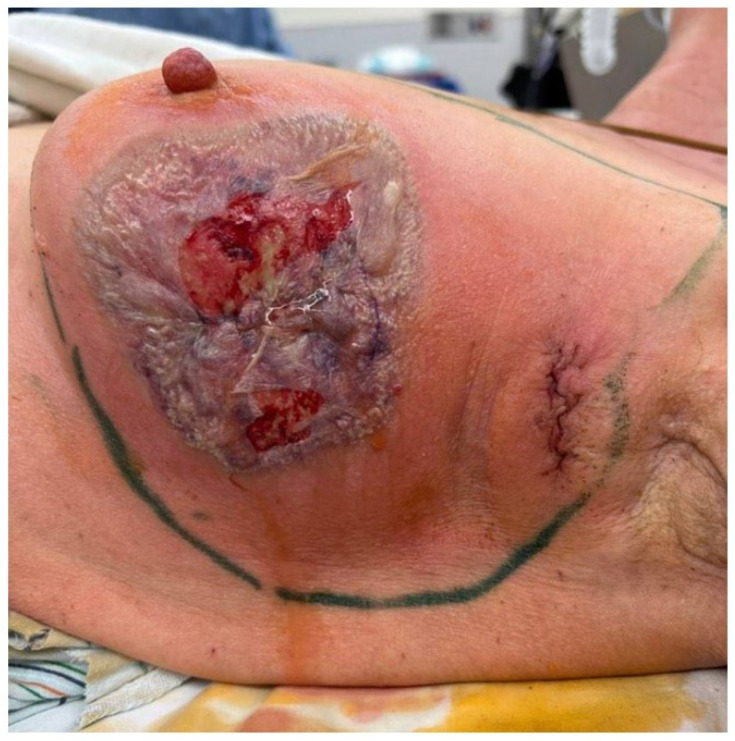
Appearance of the breast six days post-surgery showing an 8 cm violaceous, bullous plaque with surrounding erythema overlying the surgical incision. Ultrasound at the time showed a fluid collection beneath the skin.

Six days after the lumpectomy and sentinel lymph node biopsy, the patient developed worsening pain at her surgical site with local erythema and neutropenic fever. She had the rapid onset of a violaceous plaque with overlying bulla and surrounding erythema at the breast incision (Figure 1). The patient returned to the OR, at which time a 5 cm area was debrided. Tissue submitted for pathology revealed a dense neutrophilic abscess involving the dermis and subcutis with overlying ulceration. Despite initiation of broad-spectrum antibiotics and appropriate wound care, the patient’s breast and axillary wounds became increasingly necrotic, prompting additional debridement. Pathology again showed a dense neutrophilic abscess involving the dermis and subcutis with scattered foreign-body-type giant cell granulomas. Four days after the initial debridement, when cultures did not demonstrate bacterial growth, a diagnosis of pyoderma gangrenosum was made. 

**Figure 2:**
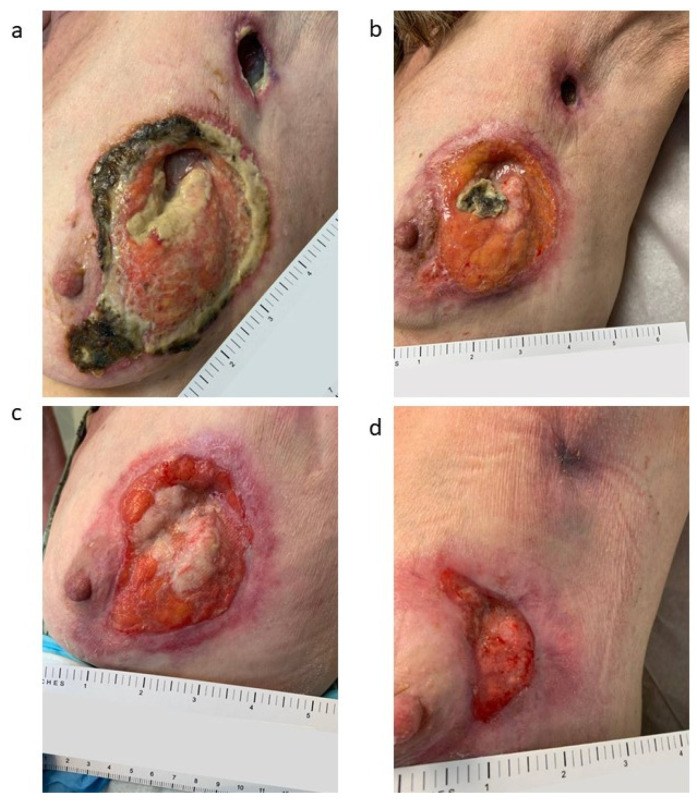
Clinical presentation in relation to chemotherapy: (**a**) Three weeks after the patient’s last debridement procedure. There is a 12 cm x 8 cm ulcerated lesion of the left lateral breast with a violaceous undermined border and areas of eschar. A separate 2.5 cm deep ulcer of the left axilla is also present. (**b**) After cycle 1 of chemotherapy. The wound is approximately 9% re-epithelialized and measures 11.4 cm × 7.6 cm. (**c**) After completion of chemotherapy. The wound is approximately 49% re-epithelialized and measures 7.6 cm × 6.4 cm. (**d**) Ten weeks after completion of chemotherapy. The left breast has a 5 cm × 2.5 cm shallow ulcerated plaque with granulation tissue and fibrinous exudate. The previously noted wound in the axilla has closed.

Given the speed of disease progression of PG and its associated morbidity, first-line therapies methylprednisolone and cyclosporine were initiated to achieve rapid disease control, and the patient was closely monitored initially in the inpatient setting during this time [12]. The benefit of short-term use of cyclosporine, particularly its ability to induce a rapid response and to improve pain with PG, was thought to outweigh the dose and time-dependent risk of malignancy progression due to immunosuppression from the drug. With therapy, the patient’s disease stabilized, but she was left with a 12 cm wound of the left lateral breast with a smaller 2.5 cm deficit in the left axilla (Figure 2a). She was discharged on prednisone, 1 mg/kg (60 mg) daily, and was seen in the wound care clinic on a weekly basis. The wound was initially managed with a TheraHoney gel and a Mepilex border. Later, Cutimed dressings were used with a Mepilex border. These dressings were chosen to promote wound healing and minimize trauma to the skin during application and removal, as such disruption might incite progression of pyoderma gangrenosum.

Given her Oncotype Dx score of 58, the patient was planned for adjuvant chemotherapy to commence one month after her last surgical debridement. Cyclosporine was discontinued and the patient received four cycles of cyclophosphamide and doxorubicin (AC) followed by four cycles of paclitaxel (T) over a period of 14 weeks. She had a moderate febrile reaction after each infusion, but wound cultures remained negative and the ulcer slowly began to form granulation tissue. A slow steroid taper was started during chemotherapy. After cycle 4 of AC, the patient developed both excruciating pain at the site of her lesion and neutropenic fever requiring hospital admission. On evaluation, she was noted to have a tender red papule on her face and an exudative lesion of the roof of her mouth. The latter was subsequently biopsied and found to also be consistent with pyoderma gangrenosum. During chemotherapy, the patient was admitted to the hospital a total of four times for management of neutropenic fever before successful completion.

To allow for maximal healing prior to the initiation of adjuvant radiation, the patient was scheduled for CT-simulation 10 weeks after her last cycle of chemotherapy (Figure 2d). Given her triple-negative grade III disease, our goal was to deliver a radiation regimen with an adequate dose and fractionation to reduce her increased risk of locoregional recurrence. As such, the standard-of-care approach to radiation was maintained without modification. The dose, target coverage, and skin dose constraints were all within standard parameters to ensure oncologic efficacy. She was planned for a total of 42.56 Gy delivered over 16 daily fractions to the whole breast, using 3D conformal radiation therapy with opposing tangent beams. A boost was not employed as the tumor bed could not be localized. 

**Figure 3:**
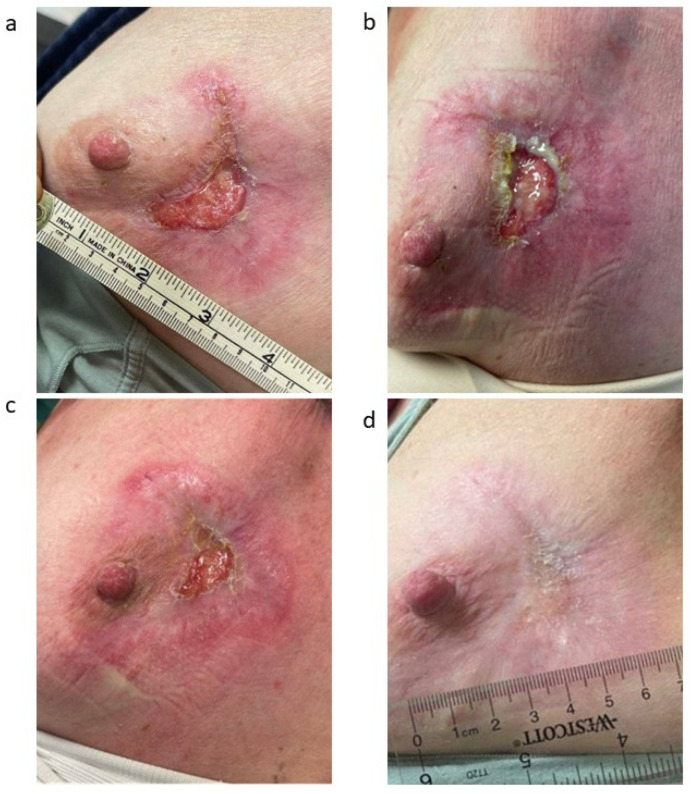
Clinical presentation during and after radiation treatment: (**a**) First on-treatment visit (13.3 Gy delivered). The lesion is approximately 95% re-epithelialized and measures 3 cm × 1.5 cm. (**b**) Second on-treatment visit (26.6 Gy delivered). The lesion now measures 2.5 cm × 1 cm. (**c**) Final on-treatment visit (39.9 Gy delivered). The lesion is approximately 98% re-epithelialized and measures 2 cm × 1 cm. (**d**) One month post treatment (42.56 Gy delivered). The lesion is completely re-epithelialized.

The patient was monitored closely during treatment with a low threshold to hold radiotherapy in case of excess skin toxicity. She was evaluated by Wound Care regularly during this period. The skin was cared for with regular application of Aquaphor and the use of silicone foam dressings to minimize wound irritation. During the course of her radiation therapy, the patient’s lesion showed significant healing. Her clinical presentation and administered radiation dose for each on-treatment visit is detailed in Figure 3. At her one-month post-treatment visit, her PG lesions had completely resolved (Figure 3d). Prednisone was discontinued and adjuvant hormonal therapy was initiated with letrozole, per the standard of care. 

## 3. Discussion

While the pathophysiology of pyoderma gangrenosum is poorly understood, it is believed to be a multifactorial combination of neutrophil dysfunction, immune system and cytokine dysregulation, and genetic predisposition [13,14]. Although this patient was known to be a BARD1 mutation carrier, BARD1 impacts homologous repair and tumor suppressor gene function. It is not known to be associated with neutrophil dysfunction [15].

Chua et al. proposed a model for neutrophil dysfunction in pyoderma gangrenosum in which uninhibited production of reactive oxygen species promotes sustained angiogenesis [16]. In their model, neutrophils with an impaired ability to suppress oxidative burst collect at the site of epithelial injury or surgical insult. These neutrophils then continue to recruit other immune cells to the site, and the resultant, sustained oxidative burst induces angiogenesis by stimulating the production of VEGF and Ang-2. The state of angiogenesis further amplifies neutrophilic chemoattractants and more dysfunctional neutrophils are recruited to the site of the lesion.

This neutrophil recruitment is central to the natural history of pyoderma gangrenosum and is driven by a complex network of proinflammatory cytokines, chemokines, and other effector molecules. Elevated levels of TNF-α, IL-1 β, IL-8, IL-17, and IL-23 have been found in PG lesions [17,18], contributing to the recruitment and activation of neutrophils and leading to tissue damage and ulcer formation. Matrix metalloproteinases (MMPs), particularly MMP-9, are also upregulated in PG [17]. This can result in excessive degradation and remodeling of extracellular matrix components, preventing the formation of stable granulation tissue and further exacerbating tissue damage. In our patient, this neutrophilic dysfunction may have contributed to her multiple aseptic febrile reactions to granulocyte colony stimulating factor (G-CSF), resulting in several hospital admissions during chemotherapy [19].

Given this natural history, immunosuppression forms the cornerstone of pyoderma gangrenosum management. Typical first-line treatments include topical or systemic corticosteroids to reduce inflammation and rapidly control lesion progression. Additional immunosuppressive agents, such as cyclosporine, are often used when corticosteroids alone are insufficient or when steroid-sparing options are preferred [20,21]. Biologic agents, such as TNF-α inhibitors (e.g., infliximab), can be employed in refractory cases of PG [22] and have shown efficacy in reducing immune-mediated tissue destruction by targeting the aforementioned inflammatory pathways.

Interestingly, studies have also demonstrated a benefit to pulsed cyclophosphamide in pyoderma gangrenosum management, particularly in severe or treatment-resistant cases. Cyclophosphamide, a potent antineoplastic and immunosuppressive drug, is an alkylating agent that targets rapidly dividing cells by causing DNA cross links and inhibiting cellular replication. In a small prospective study, nine consecutive patients with PG were treated with intravenous bolus cyclophosphamide on a monthly basis for a maximum of six doses. Complete remission, defined as 100% ulcer healing, was observed in seven patients. Partial remission and no response were each observed in one patient [23].

Due to the concern for myelosuppression and significant side effects secondary to cyclophosphamide and other such agents, their use in pyoderma gangrenosum has been limited. However, in early-stage breast cancer, as in our case report, cyclophosphamide is often used in combination with anthracyclines, taxanes, and 5-fluorouracil. In this case, the use of cyclophosphamide as adjuvant antineoplastic therapy for the patient’s breast cancer may have had the added benefit of accelerating wound healing in her PG. Its ability to target and reduce the activity of immune cells, especially the neutrophils involved in the dysregulated inflammatory response, may help control the excessive inflammation and tissue breakdown associated with this condition. This potential overlap in benefits highlights cyclophosphamide’s unique role in cases where oncologic and immunosuppressive needs intersect, suggesting that its application in cancer patients with concurrent PG could be a valuable area for further study.

While there are very few published cases documenting the effects of therapeutic radiation on active pyoderma gangrenosum, one report by Rumeileh et al. described the use of radiation in the management of refractory PG in a 32-year-old female [24]. In this case, the patient was treated with 22 separate courses of radiation to involved skin areas at single-fraction doses of between 4 Gy and 8 Gy. She achieved complete remission of all treated lesions. The authors hypothesized that radiation-induced microvascular endothelial apoptosis may have been the main driver of this outcome. This is a well-described effect of ionizing radiation [25]. Given that angiogenesis results in the amplification of dysfunctional neutrophils in PG, damage to the microvascular endothelium provided by radiation may provide benefit by disrupting further neutrophil recruitment.

Radiation has also been shown to modulate the inflammatory and immune response via the upregulation and recruitment of regulatory T cells (Tregs) [26], which function to inhibit T-cell proliferation and cytokine production. In addition, studies have shown radiation to upregulate levels of tissue inhibitors of matrix metalloproteinases (TIMPs) [27], hinting at a complex interplay that may induce a more controlled inflammatory response, shifting a wound from a chronic non-healing state to a more acute, regulated healing process. Thus, radiation might help reestablish the balance between extracellular matrix degradation and synthesis, facilitating the closure and healing of wounds in pyoderma gangrenosum patients. These effects, taken together, could break the cycle of chronic inflammation and ulceration, leading to faster wound healing.

The literature on the direct application of radiation in pyoderma gangrenosum is limited, but at minimum, our case provides evidence for the safety of chemotherapy and radiation therapy in oncologic cases complicated by it. As demonstrated by our patient’s response, PG may not be a contraindication to radiation treatment. In addition to receiving the benefit of adjuvant radiation for Stage IIB invasive ductal carcinoma, our patient demonstrated an improvement in her postoperative pyoderma gangrenosum with no adverse skin effects.

## Data Availability

The original contributions presented in this study are included in the article. Further inquiries can be directed to the corresponding author.
